# Temporal changes in gastrointestinal fungi and the risk of autoimmunity during early childhood: the TEDDY study

**DOI:** 10.1038/s41467-022-30686-w

**Published:** 2022-06-07

**Authors:** Thomas A. Auchtung, Christopher J. Stewart, Daniel P. Smith, Eric W. Triplett, Daniel Agardh, William A. Hagopian, Anette G. Ziegler, Marian J. Rewers, Jin-Xiong She, Jorma Toppari, Åke Lernmark, Beena Akolkar, Jeffrey P. Krischer, Kendra Vehik, Jennifer M. Auchtung, Nadim J. Ajami, Joseph F. Petrosino

**Affiliations:** 1 Alkek Center for Metagenomics and Microbiome Research, Department of Molecular Virology and Microbiology, Baylor College of Medicine, Houston, TX USA; 2 Department of Food Science and Technology, University of Nebraska-Lincoln, Lincoln, NE USA; 3 Translational and Clinical Research Institute, Newcastle University, Newcastle upon Tyne, UK; 4 Microbiology and Cell Science Department, Institute for Food and Agricultural Sciences, University of Florida, Gainesville, FL USA; 5 Department of Clinical Sciences, Lund University Clinical Research Center, Skåne University Hospital, Malmö, Sweden; 6 Pacific Northwest Research Institute, Seattle, WA USA; 7 Institute of Diabetes Research, Helmholtz Zentrum München, Munich, Germany; 8 Forschergruppe Diabetes, Technische Universität München, Klinikum Rechts der Isar, Munich, Germany; 9 Forschergruppe Diabetes e.V. at Helmholtz Zentrum München, Munich, Germany; 10 Barbara Davis Center for Childhood Diabetes, University of Colorado, Aurora, CO USA; 11 Center for Biotechnology and Genomic Medicine, Medical College of Georgia, Augusta University, Augusta, GA USA; 12 Institute of Biomedicine, Research Centre for Integrative Physiology and Pharmacology, University of Turku, Turku, Finland; 13 Department of Pediatrics, Turku University Hospital, Turku, Finland; 14 Department of Clinical Sciences, Lund University/CRC, Skane University Hospital, Malmö, Sweden; 15 National Institute of Diabetes & Digestive & Kidney Diseases, Bethesda, MD USA; 16 Health Informatics Institute, Morsani College of Medicine, University of South Florida, Tampa, FL USA; 17 Nebraska Food for Health Center, University of Nebraska-Lincoln, Lincoln, NE USA; 18 Program for Innovative Microbiome and Translational Research, Department of Genomic Medicine, The University of Texas MD Anderson Cancer Center, Houston, TX USA; 19 Present Address: Jinfiniti Precision Medicine, Inc, Augusta, GA USA

**Keywords:** Fungal ecology, Intestinal diseases, Autoimmune diseases

## Abstract

Fungal infections are a major health problem that often begin in the gastrointestinal tract. Gut microbe interactions in early childhood are critical for proper immune responses, yet there is little known about the development of the fungal population from infancy into childhood. Here, as part of the TEDDY (The Environmental Determinants of Diabetes in the Young) study, we examine stool samples of 888 children from 3 to 48 months and find considerable differences between fungi and bacteria. The metagenomic relative abundance of fungi was extremely low but increased while weaning from milk and formula. Overall fungal diversity remained constant over time, in contrast with the increase in bacterial diversity. Fungal profiles had high temporal variation, but there was less variation from month-to-month in an individual than among different children of the same age. Fungal composition varied with geography, diet, and the use of probiotics. Multiple *Candida* spp. were at higher relative abundance in children than adults, while *Malassezia* and certain food-associated fungi were lower in children. There were only subtle fungal differences associated with the subset of children that developed islet autoimmunity or type 1 diabetes. Having proper fungal exposures may be crucial for children to establish appropriate responses to fungi and limit the risk of infection: the data here suggests those gastrointestinal exposures are limited and variable.

## Abstract (web summary)

Here, via metagenomics and ITS2 sequencing analysis of children's stool samples from three months to four years, the authors show that the fungal composition changes and relative abundance increases at weaning, but unlike bacteria, the overall levels of fungal diversity do not change substantially over time.

## Introduction

In healthy adults, fungi are typically present at low levels in the lower gastrointestinal tract, with high temporal and interindividual fungal variation^1,2^. Fungal species can be pathogenic when the immune system is compromised or the bacterial community is altered. For instance, fungi in the gastrointestinal tract have been implicated in inflammatory bowel disease^3,4^, pancreatic cancer^5^, and invasive infections^6,7^. Due to differential fungal sequence abundance in disease cohorts relative to healthy controls, fungi have been suggested to play a role in many more diseases, including type 1 diabetes^8^.

While a considerable number of studies have examined the fungi in gastrointestinal tracts of adults, there is relatively little known about fungi found in children, where a developing immune system and immature bacterial community could allow fungi to have a greater influence^9^. Early studies primarily isolated *Candida* species from the stool of children^10^, while more recent sequencing has detected different predominant fungal taxa^11,12^. However, there has been no large-scale study to characterize changes in gastrointestinal fungi throughout early childhood.

The Environmental Determinants of Diabetes in the Young (TEDDY) study represents an ideal cohort to examine longitudinal changes in the taxonomic composition of fungi in the gut and how this relates to environmental variables. Clinical research centers in Finland, Germany, Sweden, and the United States (Colorado, Florida/Georgia, and Washington) collected monthly stool samples from three months of life in children with increased human leukocyte antigen (HLA) genetic risk for type 1 diabetes (T1D; 89%) or first-degree relative(s) with the disease (11%). Analysis of bacterial community composition in these children has linked bacterial profiles to breastfeeding and other variables but possessed no consistent signature for T1D^13,14^. Previous studies have suggested that T1D patients have atypical gastrointestinal fungal communities^8,15–17^ though none examined fungi in children before they were diagnosed with T1D.

Here, we analyze fungi in the stool of children from 3 to 48 months old using second ribosomal internal transcribed spacer (ITS2) amplicon sequencing (*n* = 845 children and 11,778 samples) and metagenomic sequencing (*n* = 888 children and 12,262 samples) to determine (1) how fungal abundance, diversity, individual variation, and taxonomic representation change over time, (2) connections between fungal profiles and environmental variables, and (3) the possible influence of fungi on the onset of autoimmunity and diseases of early childhood, such as T1D.

## Results and discussion

### Metagenomic fungal relative abundance

**Fig. 1:**
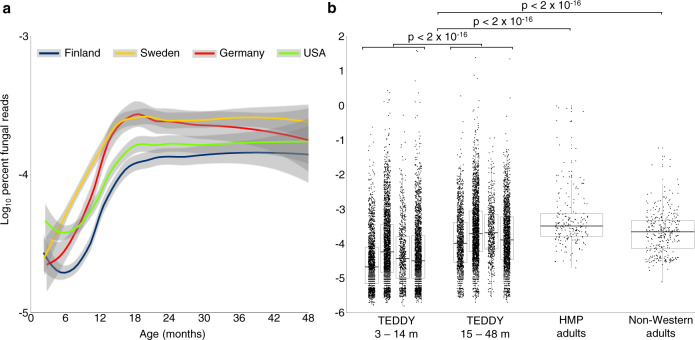
Fungal exposure increases with age. **a** Abundance of fungal reads in TEDDY metagenomic samples (*n* = 12,262 from 888 children; median 2.6 × 107 total reads/sample). Curves show LOESS fit for samples from Finland (blue; *n* = 2855 from 238 children), Sweden (yellow; *n* =  4360 from 302 children), Germany (red; *n* = 1228 from 86 children), and USA (green; *n* = 3819 from 262 children). Shaded regions represent 95% confidence intervals. **b** The fungal percent of metagenomic reads in young children (TEDDY, 3–14 m, *n* = 6547), older children (TEDDY, 15 48 m, *n* = 5621), USA adults (HMP, *n* = 203), and non-Western adults from Fiji, Madagascar, Peru, and Tanzania (*n* = 315). Displayed are the interquartile range (IQR; boxes), median (line), and 1.5 IQR (whiskers). TEDDY countries left to right are Finland (blue; *n* = 2835 from 238 children), Sweden (yellow; *n* = 4331 from 302 children), Germany (red; *n* = 1220 from 86 children), and USA (green; *n* = 3784 from 262 children). Samples with zero fungal reads are shown having one read to allow logarithmic scaling. The *P* values were calculated by Kruskal–Wallis rank-sum tests. Source data are provided in the Source Data file.

In the TEDDY nested case–control (NCC), metagenomic reads that matched fungi composed 0.014% of all reads analyzed, with relative abundances ranging from no fungal matches (7% of samples) to a maximum of 39% of reads matching fungi (Supplementary Data 1). Overall, fungi were in samples at a median of one in every 1.4 million reads (7.0 × 10^−5^% fungal reads/sample). Yet for 60 samples (from 53 children) fungi composed >0.1% of the reads. Across clinical centers, fungal abundance was lower in the first 15 m (3.6 ×  10^−5^% for 3–14 m vs 1.5 × 10^−4^% for 15–48 m; *P* < 0.001; Fig. 1a). For comparison, we used the same approach to analyze metagenomic samples from healthy U.S. adults in the Human Microbiome Project (HMP) and non-Western adults from Fiji, Madagascar, Peru, and Tanzania (Fig. 1b). While the relative abundance of fungal reads in adults was also low (HMP median 3.6 × 10^−4^%; non-Western median 2.2 × 10^−4^%), adults had a higher relative abundance of fungi than children (TEDDY median 7.2 × 10^−5^%; vs HMP or non-Western: *P* < 0.001). Multiple factors influence the interpretation of fungal abundance, such as increased genome size, uneven DNA extraction efficiency, and increased physical size. However, the very low overall amount of fungi detected here suggest they have a limited ability to influence functional aspects of the developing gut microbiome, though periodic exposure to higher levels of fungi could strongly impact future immune responses.

### Fungal diversity and taxonomy

**Fig. 2:**
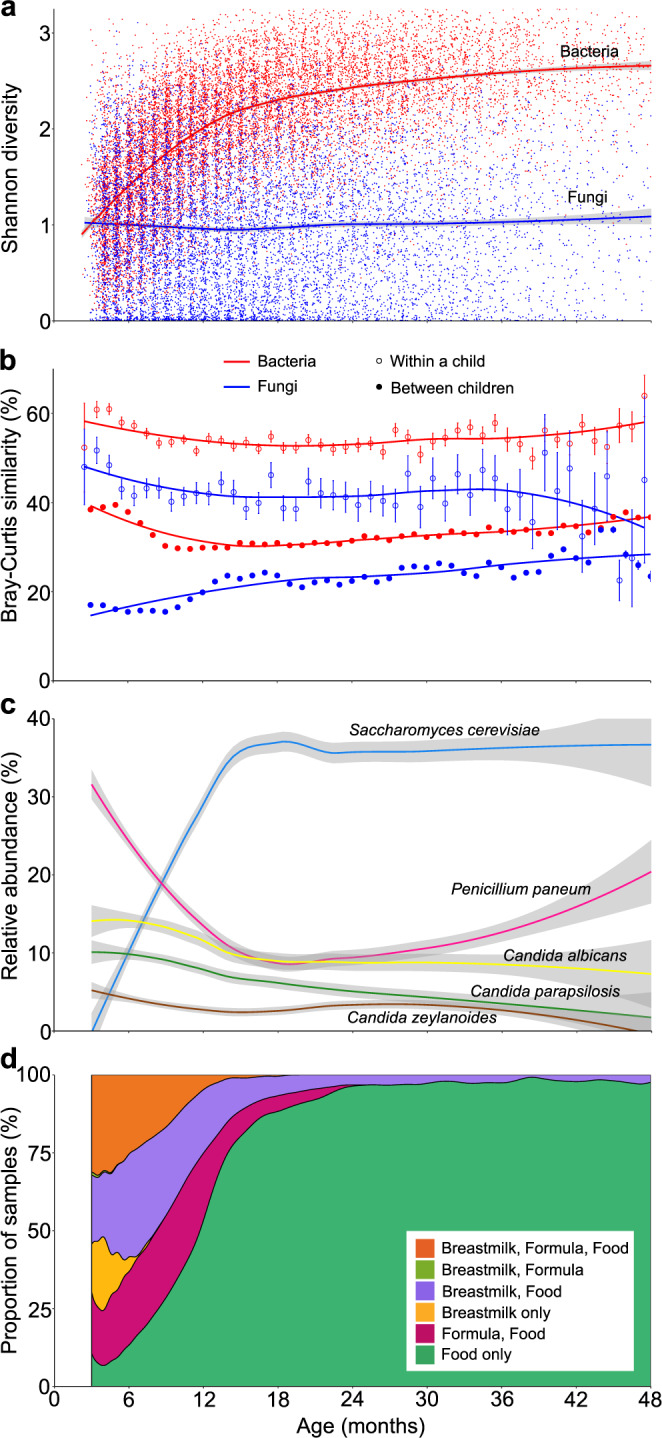
Fungal diversity, similarity, and taxonomic patterns from 3 to 48 months of life, and the corresponding dietary trends. **a** Shannon diversity of fungal ITS2 (blue; *n* = 9547 from 825 children) and bacterial 16S rRNA gene (red; *n* = 12,616 from 910 children) OTUs clustered at 97% identity and rarefied to 3000 reads/sample. Curves show LOESS fit for the data, and shaded regions represent 95% confidence intervals. **b** Mean Bray–Curtis similarity of the collection of fungal ITS2 (blue; *n* =  9547 from 825 children) and bacterial 16S rRNA gene (red; *n* = 12,616 from 910 children) OTUs clustered at 97% identity and rarefied to 3000 reads/sample, between children and within a child. Error bars display the standard error. **c** Five most abundant overall fungal taxa from 3 to 48 months as detected by analysis of ITS2 OTUs clustered at 99% identity and rarefied to 3000 reads/sample (*n* = 9547 from 825 children). Curves show LOESS fit for *Saccharomyces cerevisiae* (blue), *Penicillium paneum* (pink), *Candida albicans* (yellow), *Candida parapsilosis* (green), and *Candida zeylanoides* (brown). Shaded regions represent 95% confidence intervals. **d** Breastmilk, formula, and/or food consumption status of children by age (*n* = 12,127 from 845 children). Source data are provided in the Source Data file.

The diversity of the bacterial community (*n* = 12,616 samples from 910 children) increased over time (particularly through 15 m; ≤ vs >16 m *P* < 0.001), whereas fungal diversity was remarkably consistent, with a mean value of 1.0 on the Shannon diversity index (Fig. 2a; Pearson's *r* = 0.02, ≤ vs >16 m *P* = 0.14). There was a slight increase in the number of observed fungal operational taxonomic units (OTUs) over time, from a mean of 11.6 (SD 7.8) at 3 m to 16.7 (SD 11.0) at 48 m (Supplementary Fig. 1; ≤ vs >15 m *P* < 0.001). Fungal diversity may be steady even earlier than examined here, as was previously reported in the first month of life^18^. This dramatic difference from the increasing diversity of bacteria may reflect the distinct behavior of fungi in the gut ecosystem.

The temporal composition of fungi in a child was more similar to itself from month-to-month than to the fungi from other children of the same age (*P* < 0.001 for 3–36 m; Fig. 2b). While the similarity of fungal profiles within and between children was relatively steady over time (similar to bacteria), the fungi in an individual's stool became slightly more similar to others from the same age group between 3 and 15 m (increasing from 17% to 23% Bray–Curtis similarity), whereas the bacterial community became less similar to those of other individuals (decreasing from 38 to 31% similarity). Greater inter- than intraindividual fungal diversity has been reported in newborns^18^. However, this finding in children contrasts with observations from adults, where fungal profiles from longitudinal samples of a given subject were not significantly more similar to each other than to those of other subjects^1^. Perhaps this difference is due to the increased diversity of foods (each associated with unique fungi) consumed by adults^2^.

Overall, the most abundant fungal species detected in the stool of TEDDY children were *Saccharomyces cerevisiae*, *Penicillium paneum*, and three species of *Candida*: *C. albicans*, *C. parapsilosis*, and *C. zeylanoides* (Fig. 2c). The taxonomic composition found by analysis of metagenomic data was similar (Supplementary Data 2), but we focused on ITS2 sequence data because it benefits from both greater fungal sequence depth and a more comprehensive database for analyses. From 3 to 15 m of age, there was a considerable increase in *S. cerevisiae* and decrease in *P. paneum*. After 15 m, the relative abundance of *S. cerevisiae* was stable, while *P. paneum* gradually increased. *Candida* species gradually decreased across the entire period. The change in relative abundance of these and many other taxa corresponded to the increased fraction of children over time that consumed food and were weaned of breastmilk and/or formula (Fig. 2d). *S. cerevisiae* and *Candida* spp. are commonly found as abundant taxa in studies of gut fungi^11,12,18–20^. *Saccharomyces* is in or on some foods commonly eaten by children, such as bread and crackers. Several species of the polyphyletic genus *Candida* were among the most abundant taxa detected in TEDDY children. *C. albicans* and *C. parapsilosis* are commonly found associated with body sites, including the skin, vagina, and mouth^18,21–23^, where their presence in saliva can lead to an accumulation in the gut^2^. *C. zeylanoides* can be isolated from many foods and drinks^24,25^. Studies on different human populations have identified a range of other fungal taxa abundant in stool, including *Leptosphaerulina*^19^, Dothideomycetes^20^, and *Debaryomyces hansenii*^11^. However, the identification of elevated *Penicillium paneum* is unprecedented. Analysis of ITS2 controls and metagenomic data from a similar cohort suggest that *P. paneum* was likely an analysis pipeline contaminant that was detected only due to the extremely low abundance of fungi in many samples (Supplementary Note 1, including Supplementary Fig. 8 and Supplementary Data 8).

Comparing the taxa detected in children of the TEDDY NCC with those from adults of the HMP (*n* = 310), 367 of the combined 3900 taxa detected in the studies had significant differences in abundance (*q* < 0.2 by Kruskal–Wallis test; Supplementary Data 3a). Among the most abundant species, *P. paneum* and *Candida* spp. were significantly higher in children (Supplementary Fig. 2). Conversely, children had lower amounts of *Malassezia restricta* (*q* = 3.6 × 10^−79^); members of this genus are at the highest levels in sebaceous skin sites^26^. Until puberty, children have low secretion rates of oily sebum^27^, possibly limiting the growth of *Malassezia*. In addition, children had lower amounts of the common mushroom *Agaricus bisporus* (*q* = 8.5 ×  10^−195^) and numerous fungal pathogens of plants (e.g., *Sarocladium*, *q* = 1.8 × 10^−112^; *Tilletia*, *q* = 1.6 × 10^−79^; and *Stenocarpella maydis*, *q* = 1.0 × 10^−54^*)* that may be consumed more frequently in a typical adult diet. Since the fungi-targeting ITS2 primers also amplified plant ITS2 sequences, we observed that the composition of plant DNA also changed with the age of the children (Supplementary Fig. 3 and Supplementary Data 3b).

### Influences on fungal composition

We examined associations between fungal relative abundance, alpha diversity, beta diversity, or specific taxa and the patient's personal and clinical data (Supplementary Data 4). Several variables were associated with fungal relative abundance, including breastfeeding status, sex of child, use of probiotics, diet, and asthma status (Supplementary Data 4a and Supplementary Fig. 4). Children that stopped breastfeeding (6–15 m), had ever taken probiotics (3–21 m), were male (6–42 m), or consumed specific foods (e.g., seafood 7–18 m) had an increased relative abundance of fungi in their stool. Moreover, children that developed asthma (*n* = 76) had a decreased relative abundance of fungi at 3–7 m, but an increased relative abundance of fungi at 27–45 m. No variables consistently differed according to alpha diversity (Supplementary Data 4b). However, baby food, cow's milk, and milk product consumption corresponded to significant differences in beta diversity at 3–6 m, 7–10 m, and 11–14 m (Supplementary Data 4c). Although the proportion of children weaned of breastmilk and/or formula corresponded to the increase in *S. cerevisiae* and decrease in *P. paneum*, breastmilk, formula, or food consumption did not have statistical significance (Supplementary Data 4d). *P. paneum* and *M. restricta* at 3–6 m, and *Saccharomyces cerevisiae* at 11–14 m were significantly increased in correspondence with the bacterial communities that predominate at those time points. However, there was no correlation (all *r* < 0.3) between specific fungal and bacterial taxa. Children that took probiotics had differences in beta diversity from 3 to 6 m. However, there were no differences in specific taxa that corresponded with the use of probiotics. Lastly, the six clinical centers had differences in fungal diversity and specific taxa. Both weighted (Bray–Curtis) and unweighted (Sorensen) metrics were significantly different for clinical center and country for the 3–6-m window. The greatest taxonomic differences were in *Clavispora lusitaniae* (Florida/Georgia from 3 to 9 m), *Candida zeylanoides* (Florida/Georgia after 6 m), and *Geotrichum candidum* (Finland after 6 m) (Supplementary Fig. 5). Both *C. zeylanoides* and *C. lusitaniae* are commonly found associated with fermented foods, fruit, and juices, especially citrus that is common in Florida/Georgia^25,28^. *G. candidum* is found in many host- and non-host associated environments, including the fermented dairy product “viili” that is uniquely consumed in Finland^29^. Many of the associations identified here are worthy of directed future studies.

There were a number of variables where there were no significant associations with the fungi present, including living with siblings or pets, recent use of antibiotics, or recent infections. Unlike with bacteria, there were no fungal differences associated with the mode of birth. It is possible there were differences before the first TEDDY samples were collected at 3 m, as differential abundances of some *Candida* species on the skin and in the oral cavity at 1 m have been reported^18^. In addition, while we considered trends over multiple months, it is possible that differences seen at single time points were genuine, such as have been reported previously^30^.

### Connections with disease

Possible associations of microbial abundance with the development of islet autoimmunity (IA) or type 1 diabetes were examined. The TEDDY study used a 1:1 NCC design for IA (800 children and 11,339 samples) and T1D (224 children and 3962 samples)^31^. There was no difference in the fungal alpha diversity for those that developed IA or T1D as compared to their respective control: when examined in monthly windows, *P* < 0.05 for IA only at 45 m; for T1D only at 7, 12, and 32 m (Supplementary Data 4b). In addition, there were no consistent differences in beta diversity or taxa relative abundance between those that did and did not develop IA or T1D (Supplementary Data 4c, d). Conditional logistic regression modeling of the 40 most abundant fungal species identified one (*Talaromyces* sp., OR 0.97) and three (*C. albicans*, OR 1.04; *Candida pararugosa*, OR 1.11; *Penicillium olsonii*, OR 0.90) fungi significantly different (*q* < 0.05) between cases and controls in the IA (*n* = 304) and T1D (*n* = 90) NCCs, respectively. Odds ratios close to 1.0 suggest that there were only subtle differences in these taxa (Supplementary Data 5). Multiple studies have found *Candida albicans* or *Candida* spp. to be more prevalent in the stool of T1D patients^8,16,17^, possibly due to increased glucose in blood or impaired immune systems. However, in the TEDDY study, differences in the relative abundance of *C. albicans* and *Candida* (all species) were not significantly different in children that would develop T1D (*P* > 0.09 in all time windows; Supplementary Fig. 6), and overall, the data suggest that fungal taxa were not strongly related to the development of IA or T1D.

Although this study was not designed to examine relationships between fungi and other diseases, the numerous reported cases of asthma or celiac disease allowed us to explore possible fungal anomalies. A limitation is that HLA heterodimers may contribute to the clearing of fungal infections, and the TEDDY cohort represents children with primarily the HLA genotypes of DR4-DQ8, DR8-DQ4, and DR3-DQ2^32^, representing about 4% of newborns in the USA and Germany, 5.5% in Finland and 7.5% in Sweden. Within the IA NCC, there were no significant taxonomic differences in children that developed asthma. However, children later diagnosed with celiac disease were found to have an increased abundance of *Candida sake* after 6 m (13 m *q* = 0.003; 17 m *q* < 0.001) (Supplementary Fig. 7a and Supplementary Data 4d). Celiac disease is a T-cell-mediated autoimmune disorder of the small intestine that develops upon exposure to dietary gluten, and children with certain HLA genotypes are at increased risk^33^. Whereas *C. sake* relative abundance was consistently at 0.5% in children not diagnosed with celiac disease, after 12 m this species was >3% in those diagnosed with celiac disease. In addition, children that developed celiac disease were twice as likely to have at least one sample of ≥5% *C. sake* relative abundance (11/21 celiac vs 80/318 non-celiac children with ≥10 samples taken pre-diagnosis, *P* = 0.003 by one-tailed equal variance *t* test). *C. sake* strains occasionally cause infections^34–36^, but are more commonly isolated from non-host associated environments such as produce^37^ and seawater^38^. Very little has been published about fungi in the GI tracts of celiac disease patients, but increased frequency of *Candida* isolates were reported^39^. It has been proposed that gastrointestinal *C. albicans* may trigger the development of celiac disease because a protein in the *C. albicans* cell wall has regions of homology with gliadin, a major component of wheat gluten^40^. However, *C. albicans* was not elevated in the TEDDY children who developed celiac disease (Supplementary Fig. 7b), and the single available *C. sake* genome does not encode the gliadin-like protein, though it may be that *C. sake* induces immunological responses via another mechanism. In addition to analyzing the entire study population, conditional logistic regression was run on matched celiac disease autoimmunity (CDA) cases and controls (67 pairs of children, 748 samples; Supplementary Data 5). This identified three taxa enriched in children that would develop CDA (*q* < 0.05 and OR of 1.06–1.14): *Meyerozyma guilliermondi, Debaryomyces hansenii*, and *Rhodotorula mucilaginosa*. These species are widespread in the environment, but also opportunistic pathogens^4,41,42^. Further studies are needed to establish whether these fungi contribute to the etiology of celiac disease.

This study has shown that fungi have a very different relationship with the developing infant gut than bacteria. The abundance and composition of fungal species changed with the transition from milk/formula to solid foods, but fungal diversity remained consistent. This might reflect that many of the detected fungi are not endemic to the gut^43^, but rather passing through via habitation of other body sites (e.g., *Candida* in the mouth^44^) or dietary consumption (e.g., *S. cerevisiae* in multiple foods^2^ and *Aspergillus* and *Penicillium* in fermented or spoiled foods^45^). This is further reflected in studies of healthy adults, where most detected fungal species may be unable to colonize^46^. Here, we saw that in young children, despite increased episodes of sickness and the presence of an immature bacterial community, there was no indication of a stable fungal community, or “mycobiome”, inhabiting the gut. Nevertheless, even nonviable and non-metabolically active fungi and their cellular components can provoke an immune response^47^, so differences in fungal exposures at a young age may have immediate and lasting immunological impacts. Whereas bacterial interactions with the gut consist of a succession of colonization, it may be that fungal interactions are a succession of exposures. It will be important to establish the source of the many fungi that are detected in the gut, in order to understand variations in anti-fungal immunity and limit fungal exposures that increase risk of disease.

## Methods

An overview of all major analyses is available in Supplementary Data 6.

### Study population

An extensive description of the TEDDY study design and methods have been previously published^13,48,49^. Briefly, there were six clinical research centers: three in Europe (Finland, Germany, and Sweden) and three in the United States (Colorado, Florida/Georgia, and Washington). Children enrolled were followed prospectively. Stool samples were collected monthly from 3 m. Parents mailed the stool containers at either ambient temperature or 4 °C with guaranteed delivery within 24 h. Eighty-nine percent of the TEDDY population did not have a family history of T1D. NCC studies (1:1) were drawn from the TEDDY cohort for IA, T1D, and CDA, which were screened for and defined as previously described^13,50^. Children were matched based on age at the time of the case seroconversion, geographical location, sex, and family history of T1D. Information about mothers, pregnancy, and birth was collected during the 3-month clinic visit by validated questionnaires. Follow-up data about the children was obtained by trained staff members during scheduled visits every three months starting at 3 m. For this analysis of fungi, metadata was available for 13,052 samples from 912 children regarding mode of birth, the infant’s 5-min Apgar score, pregnancy complications, presence of maternal diabetes, gestational age, sex, age, places lived (rural, small city, suburb, big city, multiple), height and weight (at birth and throughout), pets, siblings, month of sample, human leukocyte antigen genotype, islet autoimmunity (IA) status, T1D status, asthma status, celiac disease status, presence of other acute or chronic diseases, probiotic consumption, use of antibiotics, consumption of breastmilk, formula, and a number of foods including cow's milk, apple juice, assorted fruits, vegetables, grains, and meats, seafood, eggs, soy products, cereals, vitamins and other supplements, and 53 vitamins, minerals, carbohydrates, fats, and protein as calculated from diet and supplements.

The TEDDY study was approved by local US Institutional Review Boards and European Ethics Committee Boards, including the Colorado Multiple Institutional Review Board, Medical College of Georgia Human Assurance Committee (2004–2010), Georgia Health Sciences University Human Assurance Committee (2011–2012), Georgia Regents University Institutional Review Board (2013–2015), Augusta University Institutional Review Board (2015–present), University of Florida Health Center Institutional Review Board, Washington State Institutional Review Board (2004–2012), Western Institutional Review Board (2013–present), Ethics Committee of the Hospital District of Southwest Finland, Bayerischen Landesärztekammer (Bavarian Medical Association) Ethics Committee, Regional Ethics Board in Lund, Section 2 (2004–2012), and Lund University Committee for Continuing Ethical Review (2013–present). All parents or guardians provided written informed consent before participation in genetic screening and enrollment. There was no compensation provided to participants. The ClinicalTrials.gov identifier is NCT00279318.

### Metagenomic analysis

TEDDY metagenomic libraries were sequenced on Illumina HiSeq machines using the 2 × 100-bp paired-end read protocol, resulting in a median of 2.6 × 10^7^ reads from 12,262 samples of 888 children). HMP “Healthy Human Subjects” HiSeq metagenomic data was obtained via portal_client (https://github.com/IGS/portal_client) using a manifest generated on the HMP Data Analysis and Coordinating Center website (https://portal.hmpdacc.org). Data from other studies was obtained via NCBI SRA Run Selector. General information about samples is provided in Supplementary Table 1. Reads were analyzed by HumanMycobiomeScan^51^ using the largest fungal database (“Fungi_FULL” containing 281 genomes) that we supplemented with six additional genomes of species abundant in other ITS2 sequencing studies, plus *Penicillium paneum* (“Fungi_FULLER”; Supplementary Data 7). There was no publicly available *Candida zeylanoides* genome to include. To refine the results, a representative match from each sample/taxonomic hit were analyzed with megaBLAST, and if there were no hits, by BLASTn^52^ (v2.10.0). Only BLAST hits to accession numbers with fungi (TaxID 4751) in its taxonomic lineage were retained. For TEDDY, this resulted in a total of 4.7 × 10^7^ fungal hits/3.3 × 10^11^ reads. The accuracy of our approach is supported by the fact that the mean number of fungal reads we found in the HMP dataset was similar to what was calculated previously using a different approach (0.02% here using a single sample from 205 subjects vs. 0.01% previously determined using 472 samples from 215 subjects^1^).

To resolve the abundance of individual taxa, further refinements were necessary. While BLAST results were typically better at classifying unique sequences (because unlike HumanMycobiomeScan, results were not limited to just 288 species), BLAST results from analysis of conserved regions were biased towards fungi that are prominent in the database. Therefore, we only analyzed those hits that matched the same species by both methods. Because *P. paneum* is not in the NCBI nt database (searched by BLAST) and *C. zeylanoides* is not in the HumanMycobiomeScan database, hits for these taxa were manually examined to determine validity.

### ITS2 and 16S rRNA gene sequence analysis

Sample collection, DNA extraction, sequencing, and sequence processing of the second ribosomal internal transcribed spacer (ITS2) and the 16S rRNA gene were performed as previously described^1,13^. Briefly, DNA was extracted from stool using MO BIO PowerMag Microbiome DNA isolation kits. DNA was amplified using primers ITS3F and ITS4R (targeting ITS2 of fungi, previously recommended as the best region^53^) and 515F and 806R (targeting the bacterial 16S rRNA gene V4 region) and sequenced on the Illumina MiSeq platform using the 2 × 300 bp and 2 × 250 bp kits, respectively. Merging, filtering, clustering, chimera removal, and mapping were performed using USEARCH (v7.0.1090 and 8.0.1517) and UCHIME (v4.2). The ITS2 dataset was stepwise clustered into OTUs at 97% (for analysis of diversity) and 99% identity (for taxonomic analysis). Data of 11,778 samples from 845 children were filtered to remove non-fungal sequences, then rarefied to 3000 reads/sample, resulting in 9547 samples from 825 children. Some processing of biom files, such as grouping OTUs at the species level, filtering out non-fungal taxa, rarefaction, and conversion to/from table format were conducted using MacQiime v1.8.0-20140103 (http://www.wernerlab.org/software/macqiime). The 16S rRNA gene dataset was stepwise clustered into OTUs at 97% identity. The 12,955 samples from 912 children were rarefied to 3000 reads/sample, resulting in 12,616 samples from 910 children.

Alpha diversity calculations were calculated in ATIMA (http://atima.research.bcm.edu) using 97% identity OTUs (roughly fungal species level^54^) with samples rarefied to 3000 reads. Bray–Curtis dissimilarity was calculated from 97% identity OTUs using one sample/child/month. To calculate percent similarity, values were subtracted from one and multiplied by 100. Samples were compared to either samples from other children in the given month ("between children") or to the sample from the same child in the following month (“within a child”). To identify fungal taxa more or less abundant in young children than in adults, we compared the TEDDY study with that of the HMP healthy human cohort. While samples from both studies were amplified with the same primers (ITS3-ITS4), sequenced on the same platform (MiSeq 2x300), and processed via the same pipeline, their DNA was extracted using different kits^55^, and HMP samples were collected from just one country (USA). All ages of TEDDY samples (75–2083 days, median 436) were used to compare with HMP (ITS2 sequences available at NCBI: BioProject PRJNA356769). For this analysis, raw sequences from both projects were processed and clustered together (stepwise to 99%), and the taxonomic names were merged. To visualize the changes in bacterial, fungal, and plant communities over time, biom files with bleedover reads (<10) removed and rarefied to 3000 (bacteria and fungi) or 100 (plants) reads/sample were combined by month, then group samples were rarefied to that of the month with the lowest number of samples.

Analyses were run to examine associations with alpha diversity, beta diversity, or specific taxa. Computing and graphics with R were conducted using v3.6.2 and 3.3.3, RStudio v1.0.143 and 1.0.153, and packages ggplot2 v3.2.1, vegan v2.5–6, phyloseq v1.30.0, tsne v0.1–3, plyr v1.8.5, scales v1.1.0, beepr v1.3, reshape2 v1.4.3, MaAsLin v0.0.4, MaAsLin2 v1.0.0, readr v2.1.1, kableExtra v1.3.4, and survival v3.2–13. To examine statistical correspondence of alpha diversity or beta diversity with metadata, tests were run independently on different age groups (just 1 sample/child) using the R functions “kruskal.test” and “envfit”, respectively. Spearman and Pearson correlations were determined in Excel. PCoA, NMDS, and t-SNE ordination was performed in R. NMDS ordinations were not used to examine associations with beta diversity because the results were artifactual: every data point except one clustered together. To identify associations of taxa with metadata, we analyzed 37 assorted variables (and randomized controls) in 1-month windows, and 17 major variables in 3-month windows, against 313 taxa (those comprising at least 0.01% of total reads) using MaAsLin or MaAsLin2^56^ in R. Taxonomic associations that were significant in either the 1-month or 3-month windows were examined manually to identify differences that were not due to either (1) very high relative abundance in a small number of samples, or (2) high variation over time relative to controls. Dirichlet Multinomial Mixtures (DMM) clustering was performed in mothur^57^ (v1.41.3). The resulting four (ITS2) and 11 (16S rRNA genes) groups were analyzed when examining associations (Supplementary Data 4). Differences in ratios of *Candida* were tested in Excel by one-tailed equal variance *t* tests.

There was also little correspondence of fungi with any of the extensive nutritional information, though perhaps that is because it was only collected by survey at monthly intervals, and specific foods or food groups are better predictors than nutrients, as has previously been seen for microbiome variation^58^. There were also no correlations of *r* ≥0.3 with the plants detected, though this may be due to bias in the method used for detecting plants.

Taxonomic correlations were calculated using FastSpar^59^ (v0.0.10), a program that infers correlation networks using sparse and compositional data. The input was a complete taxonomic table containing either the 3699 samples that were shared across rarefied fungi, bacteria, and plants (3000, 3000, and 100 reads/sample after removing possible bleedover reads of <10/taxa), or just the 9339 samples shared across fungi and bacteria.

### Conditional logistic regression

Using the 40 most abundant fungal species prior to disease diagnosis, conditional logistic regression was performed on matched case–control (1:1) pairs for the IA (304 pairs), T1D (90 pairs) and CDA (67 pairs) endpoints in each of the respective matched nested case–control studies. The CDA cases were identified in the nested case–control IA and T1D cohorts as of May 31, 2012 based on tissue transglutaminase antibody results as of August 31, 2017. In the full TEDDY cohort, there were 835 CDA cases; 1 CDA case developed both IA and T1D before he/she seroconverted to CDA; 42 CDA cases were IA; and the rest (792, 95%) were CDA only. There were 5963 CDA-free subjects; 95% of them were free of all three events (IA, T1D, and CDA). The biomarker data are available only on the subjects included in the TEDDY nested case–control design for IA or T1D. There were 67 pairs meeting the conditions described above that contained ITS2 data. The CDA cases, especially in the pairs identified from the 1 to 1 design, include a higher proportion with the co-occurrence of IA. For IA and T1D NCCs, there was an adjustment for the potential confounding variables of age at sample collection, HLA genotype, mode of delivery, and duration of breastfeeding. Fungal abundance was entered into the model as log_2_-transformed read counts, where a value of 0.0001 was added to avoid 0's. To correct for multiple comparisons, the Benjamini–Hochberg procedure was applied^60^.

### *Candida sake* genomic analysis

The genome of *C. sake* CBA6005 (NCBI accession GCA_003243815.1) was searched for *C. albicans* hyphal wall protein 1 (NCBI accession U64206.1) using various BLAST^61^ configurations.

### Quantitative PCR

SYBR Green-based quantitative PCR using primers ITS3 and ITS4 was performed as described previously^2^.

### Statistics and reproducibility

TEDDY study design was previously reported^48,49^. Statistical tests on experimental data were performed using R and Excel.

### Reporting summary

Further information on research design is available in the Nature Research Reporting Summary linked to this article.

## Supplementary information

Supplementary Information

Description of Additional Supplementary Files

Supplementary Data 1

Supplementary Data 2

Supplementary Data 3

Supplementary Data 4

Supplementary Data 5

Supplementary Data 6

Supplementary Data 7

Supplementary Data 8

Reporting Summary

## Data Availability

TEDDY microbiome ITS2, 16S rRNA gene, and metagenomic sequencing data have been deposited in the NCBI database of Genotypes and Phenotypes (dbGaP) with the primary accession code phs001442.v3.p2. Clinical metadata analyzed during the current study is available through the NIDDK Central Repository [https://repository.niddk.nih.gov/studies/teddy]. The data are available under controlled access in accordance with dbGaP and NIDDK authorization. Access can be obtained by requests at the listed websites. HMP “Healthy Human Subjects” HiSeq metagenomic data is available at the HMP Data Analysis and Coordinating Center website [https://portal.hmpdacc.org]. Metagenomic data from other studies (listed in Supplementary Table 1) is available via the NCBI SRA Run Selector [https://www.ncbi.nlm.nih.gov/Traces/study]. HMP ITS2 sequences are available at NCBI under BioProject PRJNA356769. The database used for HumanMycobiomeScan (“Fungi_Fuller”) containing 288 genomes is available in Supplementary Data 7. The data generated in this study are provided in the Supplementary Information/Source Data file. Source data are provided with this paper.

## Source data

Source Data

## Code availability

R code used to generate figures and calculate statistics, and related files are available at https://zenodo.org/record/6508549.
