# Supplementary material for: Temporal changes in gastrointestinal fungi and the risk of autoimmunity during early childhood: the TEDDY study
Source: Nat Commun. 2022 Jun 7;13:3151. doi: 10.1038/s41467-022-30686-w (PMC9174155; doi:10.1038/s41467-022-30686-w)
Supplement: Supplementary file 1 — Supplementary Information [file 41467_2022_30686_MOESM1_ESM.pdf]

Supplementary Information

Temporal changes in gastrointestinal fungi and the risk of autoimmunity during early childhood: the TEDDY study

Thomas A. Auchtung, Christopher J. Stewart, Daniel P. Smith, Eric W. Triplett, Daniel Agardh, William A. Hagopian, Anette G. Ziegler, Marian J. Rewers, Jin-Xiong She, Jorma Toppari, Åke Lernmark, Beena Akolkar, Jeffrey P. Krischer, Kendra Vehik, Jennifer M. Auchtung, Nadim J. Ajami & Joseph F. Petrosino

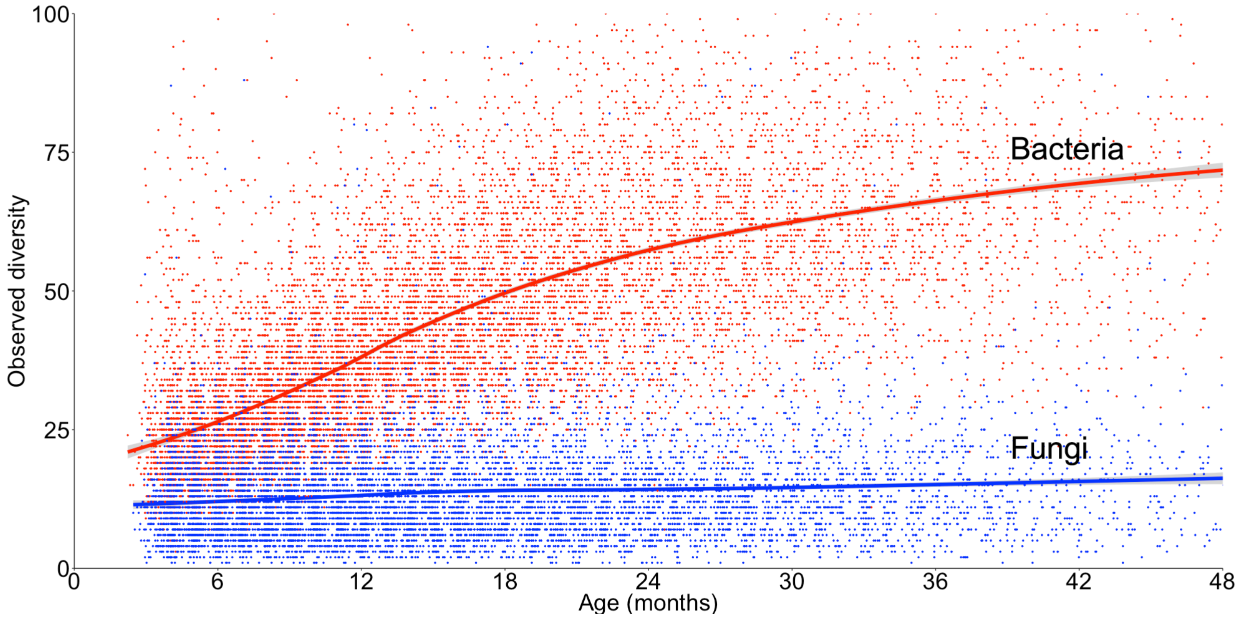

Supplementary Figure 1. Observed diversity of fungi and bacteria from 3 - 48 months of life. Curves show LOESS fit for observed ITS2 (blue, n = 9,547 from 825 children) and bacterial 16S rRNA genes (red, n = 12,616 from 910 children) clustered into OTUs at 97% identity and rarefied to 3,000 reads/sample. Shaded regions represent 95% confidence intervals. Source data are provided in the Source Data file.

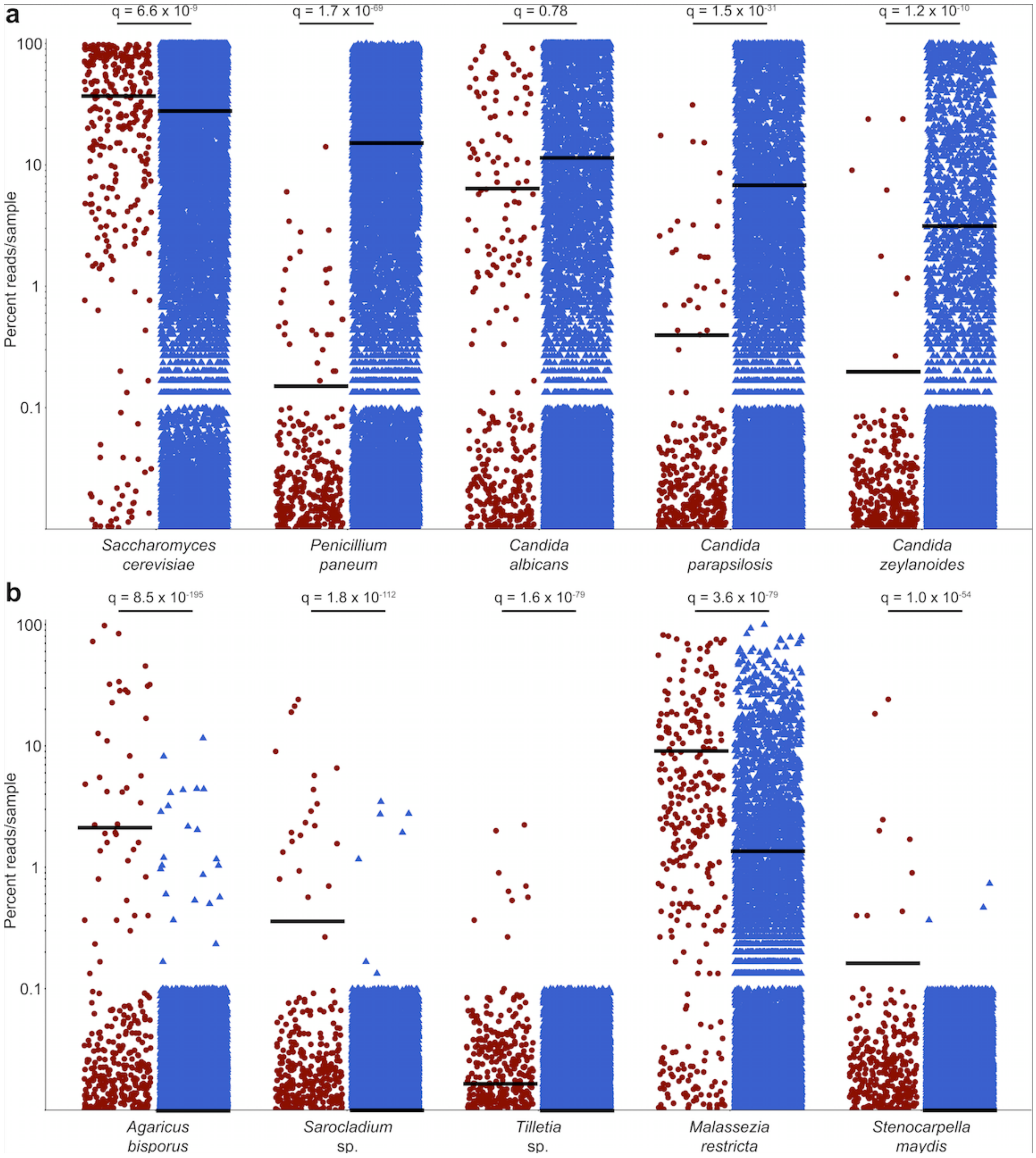

Supplementary Figure 2. Comparison of the relative abundance of select taxa in adults and children. Displayed are the relative abundance of ITS2 (clustered into OTUs at 99% identity and rarefied to 3,000 reads/sample) in HMP (n = 310 samples from 146 adults; red circles) and TEDDY (children from 2 - 68 m, n = 9,570 samples from 825 children; blue triangles) for (a) the five most abundant taxa from TEDDY, and (b) the five remaining most statistically significant differences between HMP and TEDDY. Samples with no reads for a given taxa are displayed as a random number ≤ 0.1. The mean relative abundance is marked with a black line. The q-values were determined using FDR-corrected Kruskal-Wallis rank sum tests. Source data are provided in the Source Data file.

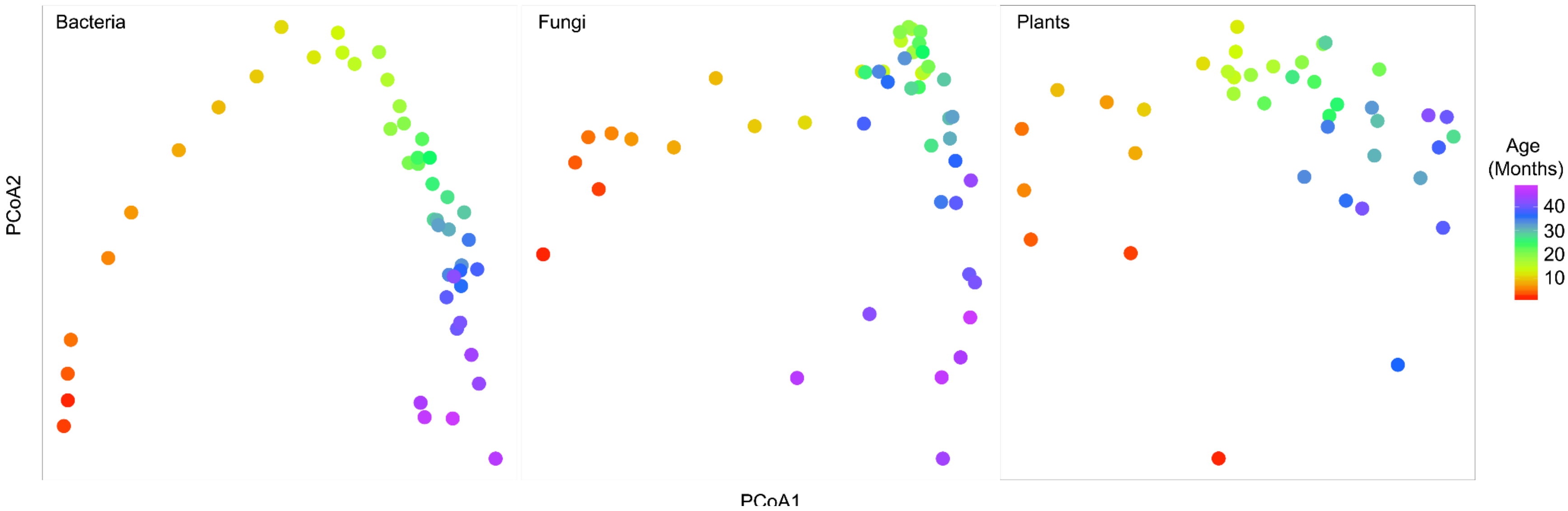

Supplementary Figure 3. Bray-Curtis PCoA ordination of the changes in bacteria, fungi, and plant community diversity over time. All rarefied individual samples (total: bacteria 16S rRNA genes (n = 12,616 from 910 children) and fungi ITS2 (n = 9,547 from 825 children): 3000 reads; plant ITS2 (n = 4,378 from 720 children): 100 reads) from a given month were merged, then community samples were rarefied to the month with the lowest total reads (bacteria: 51,000 from 2 - 48 m; fungi: 45,000 from 2 - 48 m; plant: 2,600 from 3 - 42 m). Source data are provided in the Source Data file.

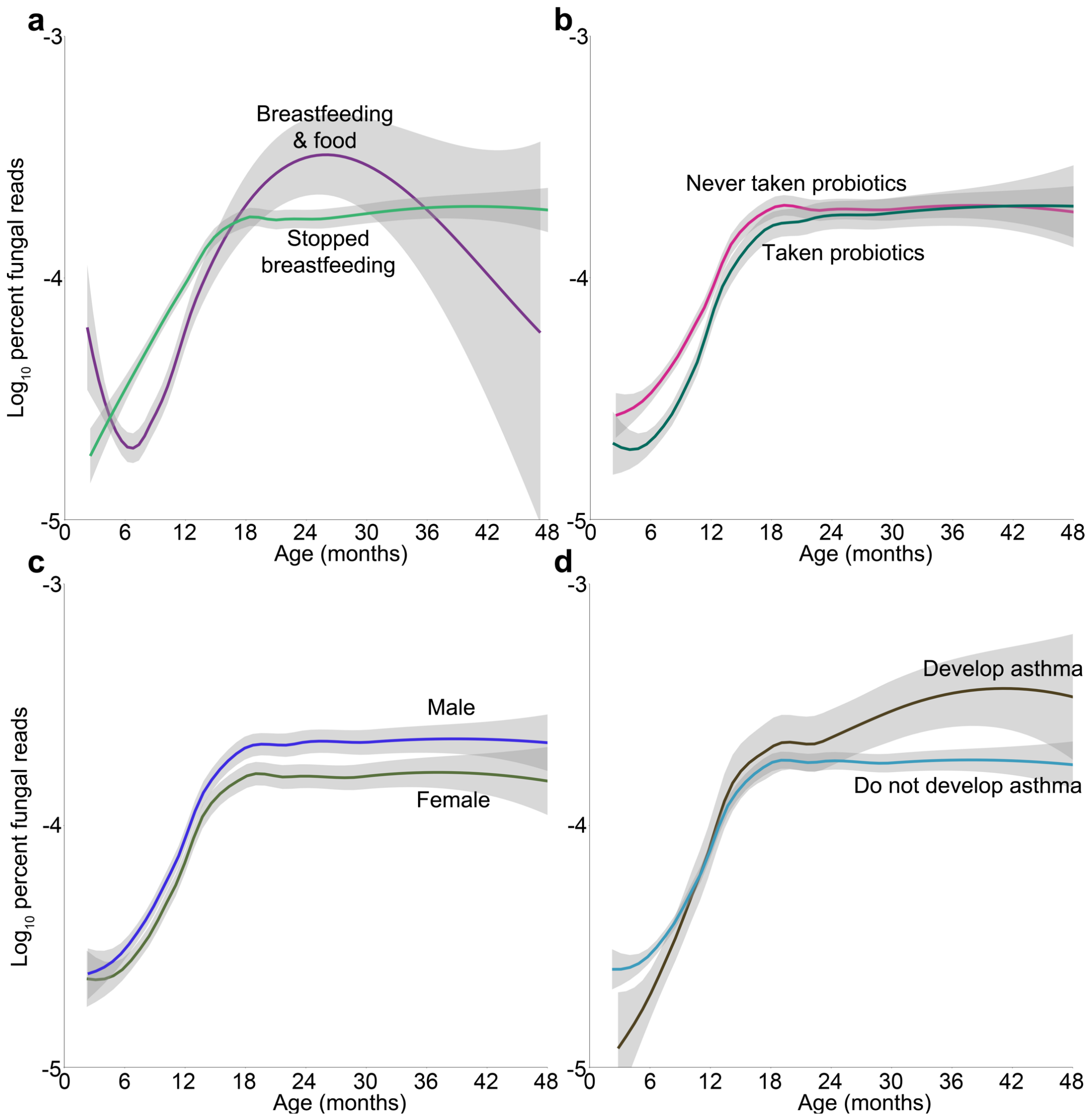

Supplementary Figure 4. Metadata that vary by fungal relative abundance. The relative abundance of fungi in metagenomic samples over time according to (a) breastfeeding status (breastfeeding and eating food, purple, n = 3,114 from 619 children; stopped breastfeeding, green, n = 8,751 from 759 children), (b) probiotics status (taken probiotics, green, n = 4,064 from 322 children; never taken probiotics, magenta, n = 8,198 from 566 children), (c) sex (male, violet, n = 6,526 from 485 children, female, green, n = 5,736 from 403 children), and (d) asthma status (develop asthma during the study, brown, n = 1,035 from 78 children; do not develop asthma, blue, n = 11,228 from 810 children). Curves show LOESS fit for the data, and shaded regions represent 95% confidence intervals. Source data are provided in the Source Data file.

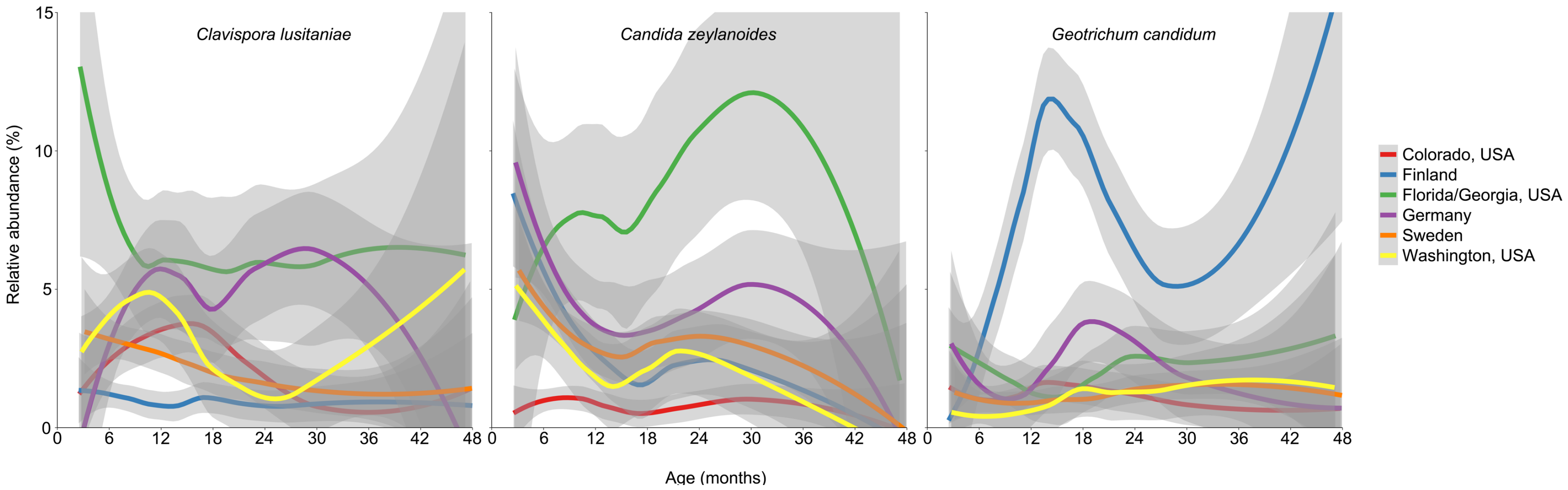

Supplementary Figure 5. Fungal species that vary by clinical center. The ITS2 relative abundance of *Clavispora lusitaniae*, *Candida zeylanoides*, and *Geotrichum candidum* over time according to location (samples/children: Colorado: 1,365/108; Finland: 1,951/203; Florida/Georgia: 655/51; Germany: 993/78; Sweden: 3,116/246; Washington: 813/77). Curves show LOESS fit for the relative abundance of ITS2 OTUs clustered at 99% identity and rarefied to 3000 reads/sample. Shaded regions represent 95% confidence intervals. Source data are provided in the Source Data file.

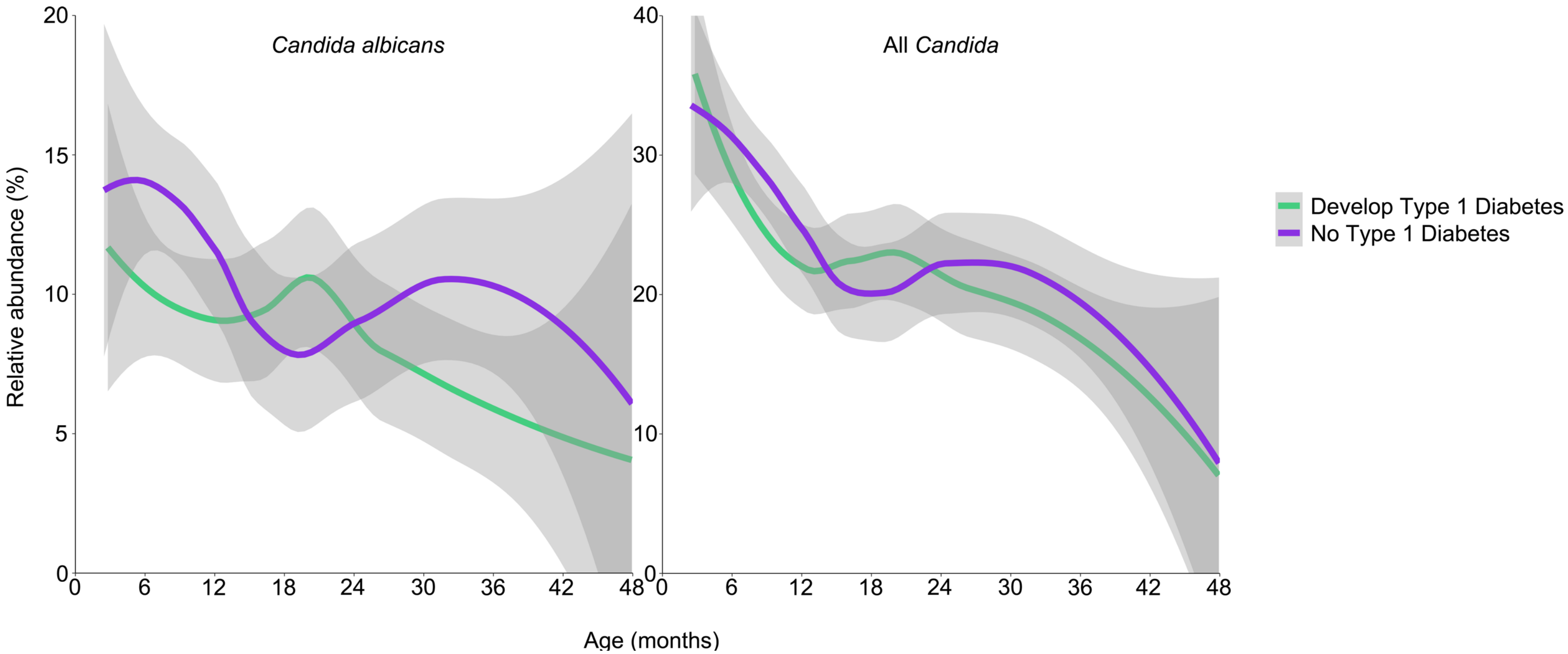

Supplementary Figure 6. Differences in *Candida albicans* and *Candida* (all species) relative abundance between matched children that do and do not develop type 1 diabetes. Curves show LOESS fit for the relative abundance of ITS2 OTUs (clustered at 99% and rarefied to 3,000 reads/sample) from 105 children that developed type 1 diabetes (n = 1,547 samples) and 103 matched controls (n = 1,400 samples) that did not. Shaded regions represent 95% confidence intervals. Source data are provided in the Source Data file.

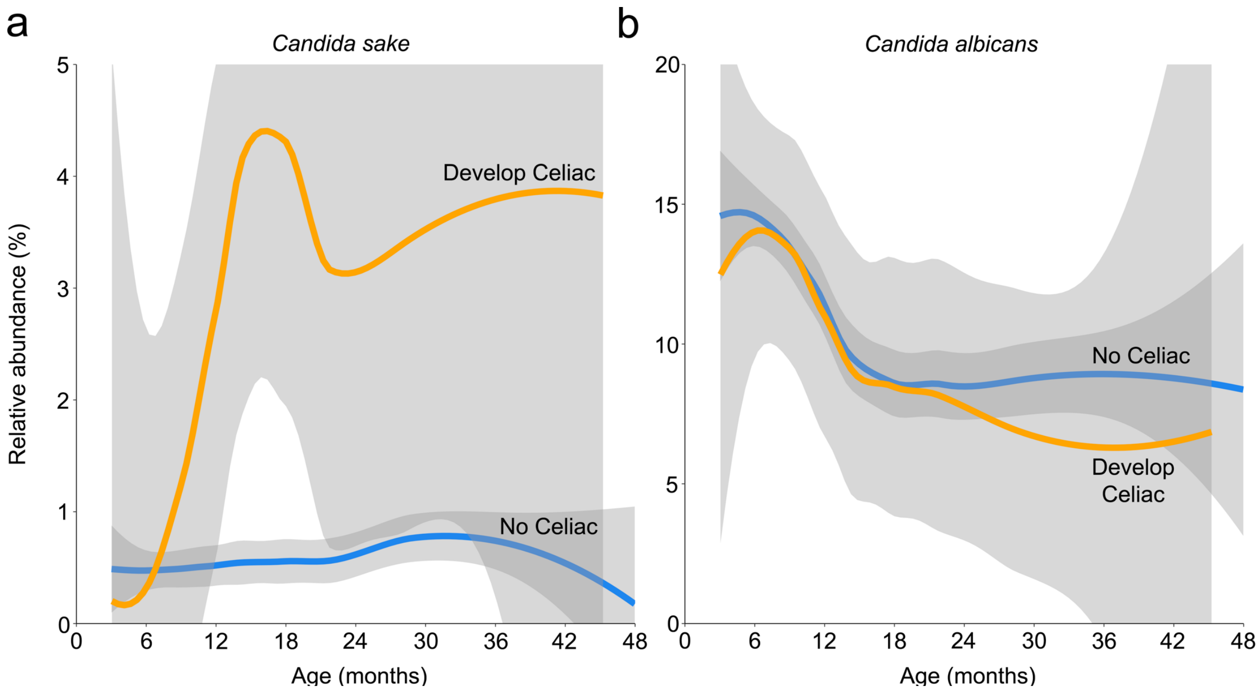

Supplementary Figure 7. Difference in *Candida sake* and *Candida albicans* relative abundance over time between children that do and do not develop celiac disease. Curves show LOESS fit for relative abundance of ITS2 OTUs (clustered at 99% and rarefied to 3,000 reads/sample) from 29 children in the IA nested case-control that developed celiac disease (yellow, n = 390 samples pre-celiac diagnosis) and 640 children (blue, n = 7,249 samples) that did not. Shaded regions represent 95% confidence intervals. Source data are provided in the Source Data file.

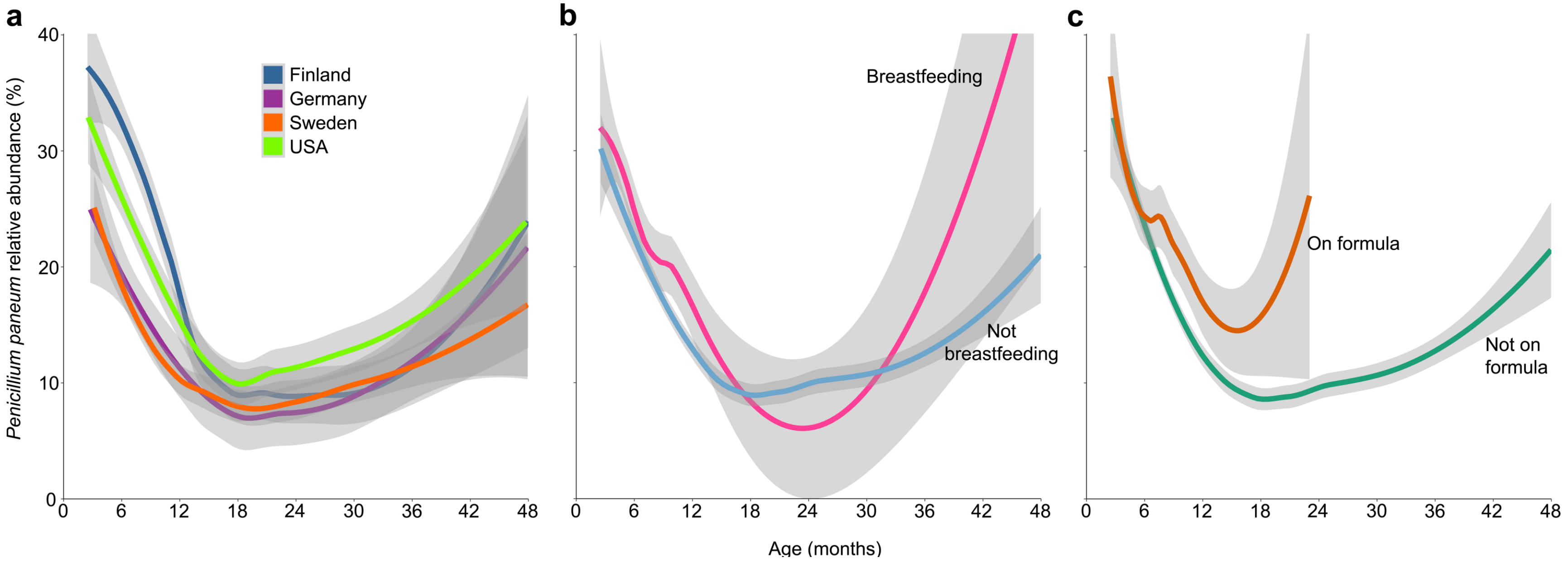

Supplementary Figure 8. Differences in *Penicillium paneum* relative abundance based on country and breastfeeding or formula feeding status. Curves show LOESS fit for relative abundance of ITS2 OTUs (clustered at 99% and rarefied to 3,000 reads/sample) of samples collected (a) from children in Finland, Germany, Sweden, or USA, (b) while children were currently breastfeeding (pink, n = 2,157 samples from 503 children) or not breastfeeding (blue, n = 6,736 samples from 660 children), and (c) while children were currently on formula (orange, n = 1,879 samples from 196 children) or not on formula (green, n = 7,697 samples from 689 children). Shaded regions represent 95% confidence intervals. Source data are provided in the Source Data file.

Supplementary Table 1. Metagenomic data analyzed.

Study	Subjects	Samples	Reads/Sample (Median)	Age	Countries	Source
TEDDY	888	12,168	2.6×10^7	3 - 48 m	USA, Finland, Sweden, Germany	dBGaP: phs001442.v3.p2
HMP[1]	203	203	3.6×10^7	adults	USA	https://portal.hmpdacc.org
MAHERY[2]	109	109	2.3×10^7	adults	Madagascar	BioProject PRJNA485056
Obregon-Tito et al.[3]	18	34	3.4×10^6	adults	Peru	BioProject PRJNA268964
Rampelli et al.[4], Smits et al.[5]	53	53	5.2×10^6	adults	Tanzania	BioProject PRJNA278393 BioProject PRJNA392180
FijiCOMP[6]	119	119	2.9×10^6	adults	Fiji	BioProject PRJNA2170522
DIABIMMUNE[7]	79	86	5.8×10^6	2 - 5 m	Finland, Estonia, Russia	BioProject PRJNA497734

Supplementary Note 1. *Penicillium paneum* background, further analysis, and discussion.

The only known report of *P. paneum* in gut samples was the isolation of two *P. paneum* colonies among 349 fungal isolates[8]. In adult HMP samples, there was no *P. paneum* reported, however sequences would have clustered into an OTU with the closely related *P. roqueforti* (2 nt difference in ITS2 region). Reanalysis of the unclustered data found that the *P. paneum* sequence in TEDDY samples was present in just two HMP subjects (each one sample), at low abundance. Differences between cohorts may result from sampling different geographic locations with local dietary characteristics. However, here we analyzed samples from multiple communities and *P. paneum* was abundant from children at all sites (Supplementary Figure 8a). The high relative abundance of *P. paneum* in the TEDDY data suggests this may either be an important group of fungi in developing children, or a contaminant indicative of low fungal DNA in samples.

P. paneum could theoretically have been detected due to its presence in consumed milk or formula. *Penicillium* has been found in mature breast milk[9], but the specific species present and their abundances have not been examined. *P. paneum* was abundant both in children who were breastfed and those who drank formula (Supplementary Figure 8b, c). Although some *Penicillium* require only very low oxygen concentrations for growth[10] and therefore might be able to colonize a developing child's GI tract, previous isolates of *P. paneum* did not grow at 37°C[11]. Therefore, like fungi in healthy adults[12], *P. paneum* detected in stool may simply be transiting, rather than colonizing, the GI tract.

Another possibility is that *P. paneum* is a contaminant of the collection/extraction/amplification/sequencing pipeline, and its presence was detected in samples with especially low fungal DNA concentrations. Unfortunately, no negative controls were performed specifically for the fungal analysis portion of the TEDDY study. Reads matching *P. paneum* were roughly equal across sequencing pools (Supplementary Data 8a) and *P. paneum* was not present in PCR positive controls (Supplementary Data 8b). However, the positive controls used relatively high concentrations of fungal template compared to the amount typically found in stool samples. *P. paneum* also had high relative abundance in the metagenomic data, suggesting this species was not solely a contaminant of PCR reagents. In a subset of samples ($n = 13$) that were known to contain a high relative abundance of *P. paneum* (>90%), copies of ITS2 were at the limit of detection (Supplementary Data 8c). Finally, we examined metagenomic samples of children aged 2 - 5 m from a similar cohort, and found zero reads matching *P. paneum* (DIABIMMUNE study, 6.1×10^8 reads examined; Supplementary Data 8d). In the TEDDY study, fungi were present in especially low abundance in the youngest samples where *P. paneum* relative abundance was highest. It may be that when there was little or no fungi present in a sample, the contaminant was detected.

Supplementary References

1. Nash, A. K. *et al.* The gut mycobiome of the Human Microbiome Project healthy cohort. *Microbiome* (2017) doi:10.1186/s40168-017-0373-4.
2. Pasolli, E. *et al.* Extensive Unexplored Human Microbiome Diversity Revealed by Over 150,000 Genomes from Metagenomes Spanning Age, Geography, and Lifestyle. *Cell* (2019) doi:10.1016/j.cell.2019.01.001.
3. Obregon-Tito, A. J. *et al.* Subsistence strategies in traditional societies distinguish gut microbiomes. *Nat. Commun.* (2015) doi:10.1038/ncomms7505.
4. Rampelli, S. *et al.* Metagenome Sequencing of the Hadza Hunter-Gatherer Gut Microbiota. *Curr. Biol.* (2015) doi:10.1016/j.cub.2015.04.055.
5. Smits, S. A. *et al.* Seasonal cycling in the gut microbiome of the Hadza hunter-gatherers of Tanzania. *Science.* (2017) doi:10.1126/science.aan4834.
6. Brito, I. L. *et al.* Mobile genes in the human microbiome are structured from global to individual scales. *Nature* (2016) doi:10.1038/nature18927.
7. Vatanen, T. *et al.* Genomic variation and strain-specific functional adaptation in the human gut microbiome during early life. *Nat. Microbiol.* (2019) doi:10.1038/s41564-018-0321-5.
8. Strati, F. *et al.* Age and gender affect the composition of fungal population of the human gastrointestinal tract. *Front. Microbiol.* **7**, 1–16 (2016).
9. Boix-Amorós, A. *et al.* Mycobiome profiles in breast milk from healthy women depend on mode of delivery, geographic location, and interaction with bacteria. *Appl. Environ. Microbiol.* (2019) doi:10.1128/AEM.02994-18.
10. Boysen, M., Skouboe, P., Frisvad, J. & Rossen, L. Reclassification of the Penicillium roqueforti group into three species on the basis of molecular genetic and biochemical profiles. *Microbiology* (1996) doi:10.1099/13500872-142-3-541.
11. O'brien, M. *et al.* Morphological and molecular characterisation of Penicillium roqueforti and P. paneum isolated from baled grass silage. *Mycol. Res.* (2008) doi:10.1016/j.mycres.2008.01.023.
12. Auchtung, T. A. *et al.* Investigating Colonization of the Healthy Adult Gastrointestinal Tract by Fungi. *mSphere* (2018) doi:10.1128/msphere.00092-18.

The TEDDY Study Group

Colorado Clinical Center: Marian Rewers, M.D., Ph.D., PI, Aaron Barbour, Kimberly Bautista, Judith Baxter, Daniel Felipe-Morales, Brigitte I. Frohnert, M.D, Marisa Stahl, M.D., Patricia Gesualdo, Michelle Hoffman, Rachel Karban, Edwin Liu, M.D., Alondra Munoz, Jill Norris, Ph.D., Holly O'Donnell, Ph.D., Stesha Peacock, Hanan Shorrosh, Andrea Steck, M.D., Megan Stern, Kathleen Waugh.

Finland Clinical Center: Jorma Toppari, M.D., Ph.D., PI, Olli G. Simell, M.D., Ph.D., Annika Adamsson, Ph.D., Sanna-Mari Aaltonen, Suvi Ahonen, Mari Åkerlund, Leena Hakola, Anne Hekkala, M.D., Henna Holappa, Heikki Hyöty, M.D., Ph.D., Anni Ikonen, Jorma Ilonen, M.D., Ph.D., Sanna Jokipuu, Leena Karlsson, Jukka Kero M.D., Ph.D., Jaakko J. Koskenniemi M.D., Ph.D., Miia Kähönen, Mikael Knip, M.D., Ph.D., Minna-Liisa Koivikko, Katja Kokkonen, Merja Koskinen, Mirva Koreasalo, Kalle Kurppa, M.D., Ph.D., Salla Kuusela, M.D., Jarita Kytölä, Jutta Laiho, Ph.D., Tiina Latva-aho, Laura Leppänen, Katri Lindfors, Ph.D., Maria Lönnrot, M.D., Ph.D., Elina Mäntymäki, Markus Mattila, Maija Miettinen, Katja Multasuo, Teija Mykkänen, Tiina Niininen, Sari Niinistö, Mia Nyblom, Sami Oikarinen, Ph.D., Paula Ollikainen , Zhian Othmani, Sirpa Pohjola, Jenna Rautanen, Anne Riikonen, Minna Romo, Satu Simell, M.D., Ph.D., Päivi Tossavainen, M.D., Mari Vähä-Mäkilä, Eeva Varjonen, Riitta Veijola, M.D., Ph.D., Irene Viinikangas, Suvi M. Virtanen, M.D., Ph.D.

Georgia/Florida Clinical Center: Jin-Xiong She, Ph.D., PI, Desmond Schatz, M.D., Diane Hopkins, Leigh Steed, Jennifer Bryant, Katherine Silvis, Michael Haller, M.D., Melissa Gardiner, Richard McIndoe, Ph.D., Ashok Sharma, Stephen W. Anderson, M.D., Laura Jacobsen, M.D., John Marks, DHSc., P.D. Towe.

Germany Clinical Center: Anette G. Ziegler, M.D., PI, Ezio Bonifacio Ph.D., Cigdem Gezginci, Anja Heublein, Eva Hohoff, Sandra Hummel, Ph.D., Annette Knopff, Charlotte Koch, Sibylle Koletzko, M.D., Claudia Ramminger, Roswith Roth, Ph.D., Jennifer Schmidt, Marlon Scholz, Joanna Stock, Katharina Warncke, M.D., Lorena Wendel, Christiane Winkler, Ph.D.

Pennsylvania Satellite Center: Dorothy Becker, M.D., Margaret Franciscus, MaryEllen Dalmagro-Elias Smith, Ashi Daftary, M.D., Mary Beth Klein, Chrystal Yates.

Sweden Clinical Center: Åke Lernmark, Ph.D., PI, Daniel Agardh, M.D., Ph.D., Carin Andrén Aronsson, Ph.D., Rasmus Bennet, Corrado Cilio, Ph.D., M.D., Susanne Dahlberg, Ulla Fält, Malin Goldman Tsubarah, Emelie Ericson-Hallström, Lina Fransson, Thomas Gard, Emina Halilovic, Gunilla Holmén, Susanne Hyberg, Berglind Jonsdottir, M.D., Ph.D., Naghmeh Karimi, Helena Elding Larsson, M.D., Ph.D., Marielle Lindström, Markus Lundgren, M.D., Ph.D., Marlena Maziarz, Ph.D., Maria Månsson Martinez, Jessica Melin, Zeliha Mestan, Caroline Nilsson, Yohanna Nordh, Kobra Rahmati, Anita Ramelius, Falastin Salami, Anette Sjöberg, Carina Törn, Ph.D., Ulrika Ulvenhag, Terese Wiktorsson, Åsa Wimar.

Washington Clinical Center: William A. Hagopian, M.D., Ph.D., PI, Michael Killian, Claire Cowen Crouch, Jennifer Skidmore, Luka-Sophia Bowen, Mikeil Metcalf, Arlene Meyer, Jocelyn Meyer, Denise Mulenga, Nole Powell, Jared Radtke, Shreya Roy, Davey Schmitt, Preston Tucker.

Data Coordinating Center: Jeffrey P. Krischer, Ph.D., PI, Rajesh Adusumali, Sarah Austin-Gonzalez, Maryouri Avendano, Sandra Baethke, Brant Burkhardt, Ph.D., Martha Butterworth, Nicholas Cadigan, Joanna Clasen, Kevin Counts, Laura Gandolfo, Jennifer Garmeson, Veena Gowda, Christina Karges, Shu Liu, Xiang Liu, Ph.D., Kristian Lynch, Ph.D., Jamie Malloy, Lazarus Mramba, Ph.D., Cristina McCarthy, Jose Moreno, Hemang M. Parikh, Ph.D., Cassandra Remedios, Chris Shaffer, Susan Smith, Noah Sulman, Ph.D., Roy Tamura, Ph.D., Dena Tewey, Michael Toth, Ulla Uusitalo, Ph.D., Kendra Vehik, Ph.D., Ponni Vijayakandipan, Melissa Wroble, Jimin Yang, Ph.D., R.D., Kenneth Young, Ph.D.
Past staff: Michael Abbondondolo, Lori Ballard, Rasheedah Brown, David Cuthbertson, Stephen Dankyi, Christopher Eberhard, Steven Fiske, David Hadley, Ph.D., Kathleen Heyman, Belinda Hsiao, Francisco Perez Laras, Hye-Seung Lee, Ph.D., Qian Li, Ph.D., Colleen Maguire, Wendy McLeod, Aubrie Merrell, Steven Meulemans, Ryan Quigley, Laura Smith, Ph.D.

National Institutes of Diabetes and Digestive and Kidney Diseases Project Scientist: Beena Akolkar, Ph.D.

Autoantibody Reference Laboratories: Liping Yu, M.D., Dongmei Miao, M.D., Kathleen Gillespie, Kyla Chandler, Ilana Kelland, Yassin Ben Khoud, Matthew Randell.

HLA Reference Laboratory: William Hagopian, M.D., Ph.D., Jared Radtke, Preston Tucker.
Previously Henry Erlich, Ph.D., Steven J. Mack, Ph.D., Anna Lisa Fear.

Metagenomics and Microbiome Laboratory: Joseph F. Petrosino, Ph.D., Nadim J. Ajami, Ph.D., Richard E. Lloyd, Ph.D., Matthew C. Ross, Ph.D., Jacqueline L. O'Brien, Ph.D., Diane S. Hutchinson, Ph.D., Daniel P. Smith, Ph.D., Matthew C. Wong, Xianjun Tian, Ph.D., Tulin Ayvaz, Auriole Tamegnon, Nguyen Truong, Hannah Moreno, Lauren Riley, Eduardo Moreno, Tonya Bauch, Lenka Kusic, Ginger Metcalf, Donna Muzny, HarshaVArdhan Doddapaneni, Ph.D., Richard Gibbs, Ph.D.

Repository: Sandra Ke, Niveen Mulholland, Ph.D.

Other contributors: Thomas Briese, Ph.D., Columbia University. Todd Brusko, Ph.D., University of Florida. Suzanne Bennett Johnson, Ph.D., Florida State University. Eoin McKinney, Ph.D., University of Cambridge. Tomi Pastinen, M.D., Ph.D., The Children's Mercy Hospital. Eric Triplett, Ph.D., University of Florida.
