# Supplementary material for: Temporal changes in gastrointestinal fungi and the risk of autoimmunity during early childhood: the TEDDY study
Source: Nat Commun. 2022 Jun 7;13:3151. doi: 10.1038/s41467-022-30686-w (PMC9174155; doi:10.1038/s41467-022-30686-w)
Supplement: Supplementary file 2 — Description of Additional Supplementary Files [file 41467_2022_30686_MOESM2_ESM.docx]

**Description of Additional Supplementary Files**

**File Name:** Supplementary Data 1

**Description:** Results from metagenomic analysis. (a) Fungal hits, sorted by taxonomic identity and genomic location. (b) Percent fungi per sample

**File Name:** Supplementary Data 2

**Description:** Comparison of the twenty most abundant fungal taxa detected from metagenomic and ITS2 analyses, according to the percent of subjects and percent of reads.

**File Name:** Supplementary Data 3

**Description:** Comparison of taxa detected by ITS2 analysis in TEDDY and HMP projects.(a) fungal taxa and (b) plant taxa compared by percent of samples, percent of reads, and Kruskal-Wallis testing (with and without FDR-correction) of significant differences in abundance by study.

**File Name:** Supplementary Data 4

**Description:** Associations of patient's personal and clinical data with fungal (a) relative abundance, (b) alpha diversity, (c) beta diversity, or (d) taxa. Statistical significance was assessed in R using kruskal.test (a,b), envfit (c) and MaAsLin (d).

**File Name:** Supplementary Data 5

**Description:** Results from conditional logistic regression modeling of the most abundant fungal species for IA, T1D, and CDA cases and controls. The q-value was determined using false discovery rate correction.

**File Name:** Supplementary Data 6

**Description:** Overview of all major analyses.

**File Name:** Supplementary Data 7

**Description:** List of fungal genus, species, and accession numbers in database used for metagenomic analyses.

**File Name:** Supplementary Data 8

**Description:** Further analysis Penicillium paneum. (a) P. paneum ITS2 reads by sequencing pool, (b) P. paneum in PCR positive controls, (c) quantitative PCR detection of ITS2 in samples where P. paneum had high relative abundance in ITS2 sequencing, (d) metagenomic data from a similar cohort analyzed for the presence of P. paneum.
