# Supplementary material for: Temporal changes in gastrointestinal fungi and the risk of autoimmunity during early childhood: the TEDDY study
Source: Nat Commun. 2022 Jun 7;13:3151. doi: 10.1038/s41467-022-30686-w (PMC9174155; doi:10.1038/s41467-022-30686-w)
Supplement: Supplementary file 11 — Reporting Summary [file 41467_2022_30686_MOESM11_ESM.pdf]

nature portfolio

Corresponding author(s): Thomas A. Auchtung
Joseph F. Petrosino

Last updated by author(s): Apr 30, 2022

Reporting Summary

Nature Portfolio wishes to improve the reproducibility of the work that we publish. This form provides structure for consistency and transparency in reporting. For further information on Nature Portfolio policies, see our Editorial Policies and the Editorial Policy Checklist.

Statistics

For all statistical analyses, confirm that the following items are present in the figure legend, table legend, main text, or Methods section.

n/a	Confirmed	
☐	☒	The exact sample size (n) for each experimental group/condition, given as a discrete number and unit of measurement
☐	☒	A statement on whether measurements were taken from distinct samples or whether the same sample was measured repeatedly
☐	☒	The statistical test(s) used AND whether they are one- or two-sided<br>*Only common tests should be described solely by name; describe more complex techniques in the Methods section.*
☐	☒	A description of all covariates tested
☐	☒	A description of any assumptions or corrections, such as tests of normality and adjustment for multiple comparisons
☐	☒	A full description of the statistical parameters including central tendency (e.g. means) or other basic estimates (e.g. regression coefficient) AND variation (e.g. standard deviation) or associated estimates of uncertainty (e.g. confidence intervals)
☐	☒	For null hypothesis testing, the test statistic (e.g. F, t, r) with confidence intervals, effect sizes, degrees of freedom and P value noted<br>*Give P values as exact values whenever suitable.*
☒	☐	For Bayesian analysis, information on the choice of priors and Markov chain Monte Carlo settings
☐	☒	For hierarchical and complex designs, identification of the appropriate level for tests and full reporting of outcomes
☐	☒	Estimates of effect sizes (e.g. Cohen's d, Pearson's r), indicating how they were calculated

Our web collection on statistics for biologists contains articles on many of the points above.

Software and code

Policy information about availability of computer code

Data collection	No software was used for data collection.
Data analysis	USEARCH v7.0.1090 and 8.0.1517<br>UCHIME v4.2<br>R v3.6.2 and 3.3.3<br>RStudio v1.0.143 and 1.0.153<br>mothur v1.41.3<br>MacQIIME v1.8.0-20140103<br>R package ggplot2 v3.2.1<br>R package vegan v2.5-6<br>R package phyloseq v1.30.0<br>R package tsne v0.1-3<br>R package plyr v1.8.5<br>R package scales v1.1.0<br>R package beepr v1.3<br>R package reshape2 v1.4.3<br>R package MaAsLin v0.0.4<br>R package MaAsLin2 v1.0.0<br>R package readr v2.1.1<br>R package kableExtra v1.3.4<br>R package survival v3.2-13<br>FastSpar v0.0.10

BLAST v2.10.0
HumanMycobiomeScan (no version listed)
R code is available at https://zenodo.org/record/6508549

For manuscripts utilizing custom algorithms or software that are central to the research but not yet described in published literature, software must be made available to editors and reviewers. We strongly encourage code deposition in a community repository (e.g. GitHub). See the Nature Portfolio guidelines for submitting code & software for further information.

Data

Policy information about availability of data

All manuscripts must include a data availability statement. This statement should provide the following information, where applicable:

- Accession codes, unique identifiers, or web links for publicly available datasets
- A description of any restrictions on data availability
- For clinical datasets or third party data, please ensure that the statement adheres to our policy

TEDDY ITS2, 16S rRNA gene, and metagenomic data that support the findings of this study are available in the NCBI database of Genotypes and Phenotypes (dbGaP) with the primary accession code phs001442.v3.p2, in accordance with the dbGaP controlled-access authorization process.
Clinical metadata analyzed during the current study will be made available in the NIDDK Central Repository at https://www.niddkrepository.org/studies/teddy/.
HMP 'Healthy Human Subjects' HiSeq metagenomic data is available at the HMP Data Analysis and Coordinating Center website (https://portal.hmpdacc.org).
Metagenomic data from other studies (listed in Supplementary Table 1) is available via NCBI SRA Run Selector (https://www.ncbi.nlm.nih.gov/Traces/study).
Human Microbiome Project ITS2 sequences are available at NCBI (BioProject PRJNA356769).
The database used for HumanMycobiomeScan ('Fungi_Fuller') containing 288 genomes is available in Supplementary Data 7.

Field-specific reporting

Please select the one below that is the best fit for your research. If you are not sure, read the appropriate sections before making your selection.

- [x] Life sciences
- [ ] Behavioural & social sciences
- [ ] Ecological, evolutionary & environmental sciences

For a reference copy of the document with all sections, see nature.com/documents/nr-reporting-summary-flat.pdf

Life sciences study design

All studies must disclose on these points even when the disclosure is negative.

Sample size	Longitudinal stool samples between months 3-48 of life were analyzed by sequencing of the second internal transcribed spacer region (n=11,778 samples from 845 children) and metagenomic sequencing (n=12,262 samples from 888 children). Bacterial 16S rRNA genes (12,616 samples from 910 children) were used as a comparison. The cohort is a nested case-control design.<br>Sample sizes were chosen previously to allow for in-depth study of the etiology of Type 1 Diabetes. This dataset is larger than any previous study of fungi in children and allowed for extensive, accurate analyses to be conducted.
Data exclusions	All samples with low sequencing depth were excluded (less than 3,000 ITS2 or 16S rRNA gene reads, and less than 4.6 million metagenomic reads).
Replication	Observational cohort. No replication.
Randomization	IA and T1D controls were matched individually to cases as described previously. Cases were sampled until diagnosis of T1D and matched control samples were included up until the corresponding day of life.
Blinding	There was no blinding used. TEDDY is an observational follow-up study.

Reporting for specific materials, systems and methods

We require information from authors about some types of materials, experimental systems and methods used in many studies. Here, indicate whether each material, system or method listed is relevant to your study. If you are not sure if a list item applies to your research, read the appropriate section before selecting a response.

Materials & experimental systems

n/a	Involved in the study	
☒	☐	Antibodies
☒	☐	Eukaryotic cell lines
☒	☐	Palaeontology and archaeology
☒	☐	Animals and other organisms
☐	☒	Human research participants
☒	☐	Clinical data
☒	☐	Dual use research of concern

Methods

n/a	Involved in the study	
☒	☐	ChIP-seq
☒	☐	Flow cytometry
☒	☐	MRI-based neuroimaging

Human research participants

Policy information about studies involving human research participants

Population characteristics	Children aged 3-48 m from six geographical locations (In Europe: Finland, Germany, Sweden; in the United States: Colorado, Florida/Georgia, and Washington). The cohort was at high risk for developing IA or T1D. Approximately 45% of the children were female. As this was an observational cohort, treatment was not part of the study.
Recruitment	Children were recruited based on risk for T1D, as has been extensively described previously in Krischer et al. and Rewers et al. (references 48, 49).
Ethics oversight	The TEDDY study was approved by local US Institutional Review Boards and European Ethics Committee Boards, including the Colorado Multiple Institutional Review Board, Medical College of Georgia Human Assurance Committee (2004–2010), Georgia Health Sciences University Human Assurance Committee (2011–2012), Georgia Regents University Institutional Review Board (2013–2015), Augusta University Institutional Review Board (2015–present), University of Florida Health Center Institutional Review Board, Washington State Institutional Review Board (2004– 2012), Western Institutional Review Board (2013–present), Ethics Committee of the Hospital District of Southwest Finland, Bayerischen Landesärztekammer (Bavarian Medical Association) Ethics Committee, Regional Ethics Board in Lund, Section 2 (2004–2012), and Lund University Committee for Continuing Ethical Review (2013–present). All parents or guardians provided written informed consent before participation in genetic screening and enrollment. The study was performed in compliance with all relevant ethical regulations.

Note that full information on the approval of the study protocol must also be provided in the manuscript.
